# A Review: The Triterpenoid Saponins and Biological Activities of *Lonicera Linn.*

**DOI:** 10.3390/molecules25173773

**Published:** 2020-08-19

**Authors:** Zhongying Fang, Jia Li, Ran Yang, Lei Fang, Yongqing Zhang

**Affiliations:** 1 Key Laboratory of Natural Pharmaceutical Chemistry, Shandong University of Traditional Chinese Medicine, Jinan 250355, China; 56kind@163.com (Z.F.); LJYTL7172@163.com (J.L.); cpu1045405@126.com (R.Y.); 2 School of Biological Science and Technology, University of Jinan, Jinan 250022, China

**Keywords:** *Lonicera Linn.*, saponins, biological activities

## Abstract

*Lonicera Linn.* is an important genus of the family *Caprifoliaceae* comprising of approximately 200 species, and some species of which have been usually used in traditional Chinese medicine for thousands of years. Some species of this genus can also be used in functional foods, cosmetics and other applications. The saponins, as one of most important bioactive components of the *Lonicera Linn.* genus, have attracted the attention of the scientific community. Thus, a comprehensive and systematic review on saponins from the genus is indispensable. In this review, 87 saponins and sapogenin from the genus of *Lonicera Linn.*, together with their pharmacological activities including hepatoprotective, anti-inflammatory, anti-bacterial, anti-allergic, anti-tumor, and immunomodulatory effects, and hemolytic toxicity were summarized.

## 1. Introduction

The *Lonicera Linn.* genus belongs to the family *Caprifoliaceae* comprising about 200 species spread throughout north temperate and subtropical regions around the world, which contains around 98 species spread all over its provinces with the most species in the southwest of China [1,2]. In traditional Chinese medicine, certain plants of this genus are diffusely applied in the treatment of carbuncle, swelling, furuncle, pharyngitis, erysipelas, heat toxin blood dysentery, wind-heat type common cold, and febrile disease [3,4,5,6]. Some species of this genus can also be used in functional foods, cosmetics and other applications, such as *Lonicerae japonica* Thunb. [7]. *L. japonica* Thunb., as a kind of traditional Chinese medicine for both medicine and food, can be used to make herbal tea and toothpaste and so on.

With the rapid development of separation technology and 2D NMR spectroscopy, more and more triterpenoid saponins have been isolated from the *Lonicera Linn.* genus [8,9]. Saponins constituents have not only high contents but also diverse biological activities in medicinal plants. A number of researchers have studied the saponins chemical ingredients and pharmacological properties of some species from this genus [10,11,12]. Many species have been reported to possess triterpenoid saponins constituents, such as *L. japonica* Thunb., *L. confuse* DC., *L. macranthoides* Hand.-Mazz., *L. hypoglauca* Miq., *L. maackii* Maxim., *L. saccate* Rehd., *L. gracilipes* var. glandulosa Maxim., *L. bournei* Hemsl., *L. fulvotomentosa* Hsu et S.C. Cheng, *L. nigru* L., *L. nigra*. L., *L. dasystyla* Rehd., *L. similis* Hemsl., and so on.

The phytochemical and biological activities properties of species from the *Lonicera Linn.* genus were summarized in this review. To data, 87 triterpenoid saponins and sapogenin have been isolated from the genus, which were classified to six major saponins types: Hederin-type, Oleanane-type, Ursane-type, Lupane-type, Fernane-1-type, and Fernane-2-type according to the chemical structure of sapogenin. Hederin-type, Oleanane-type and Ursane-type triterpenoid saponins were possessed 6/6/6/6/6 pentacyclic saponins skeleton, and they were very similar, except for the presence of various substituents group at C-23 and the different position of methyl group. Lupane-type, Fernane-1-type and Fernane-2-type triterpenoid saponins were possessed 6/6/6/6/5 pentacyclic saponins skeleton, and the main difference was the position of isopropyl and carbonyl moieties. Furthermore, the biological activities of those saponins compounds have been diffusely investigated, such as hepatoprotective, anti-inflammatory, anti-bacterial, anti-allergic, immunomodulatory, anti-tumor, molluscicidal, and anti-alzheimer’s disease (AD) activities, hemolytic toxicity, and so on.

It will provide the evidence for future research of the *Lonicera Linn.* genus and its active components in further pharmacological and clinical applications.

## 2. Constituents

Eighty-seven triterpenoid saponins and sapogenin compounds have been isolated from the *Lonicera Linn.* genus, including 46 Hederin-type triterpenoid saponins, 17 Oleanane-type triterpenoid saponins, 4 Ursane-type triterpenoid saponins, 8 Lupane-type triterpenoid saponins, 3 Fernane-1-type triterpenoid saponins, 2 Fernane-2-type triterpenoid saponins, and 7 other compounds. The saccharide chain is linked at the C-3 or C-28 position of the saponins, and the monosaccharides may include *β*-d-glucopyranosyl, *α*-l-arabinopyranosyl, *α*-l-rhamnopyranosyl and *β*-d-xylopyranosyl.

### 2.1. Hederin-Type Triterpenoid Saponins

**Figure 1:**
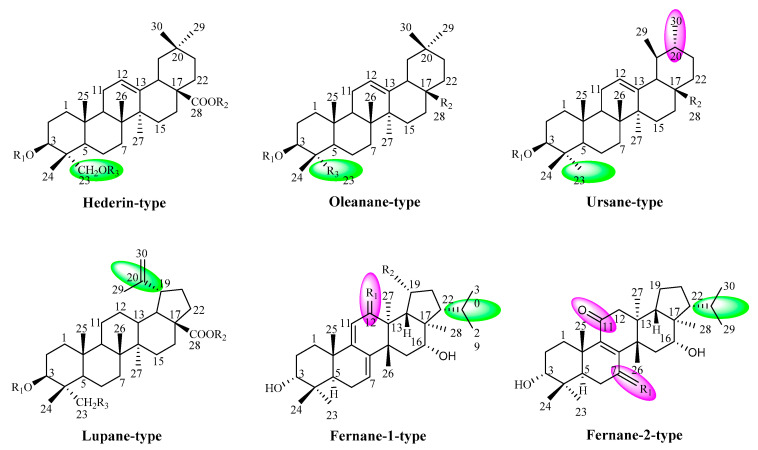
Skeleton of triterpenoid saponins from *Lonicera Linn*.

Up to now, 46 Hederin-type triterpenoid saponins (**1**–**46**) have been isolated from *L. japonica* Thunb., *L. confuse* DC., *L. macranthoides* Hand.-Mazz., *L. maackii* Maxim., *L. fulvotomentosa* Hsu et S.C. Cheng, *L. nigru* L., *L. nigra*. L. and *L. dasystyla* Rehd. species, the structures of which are shown in Figure 1 and Table 1. Cauloside A (**1**) [13], Hederagenin-3-*O*-*α*-l-rhamnopyranosyl-(1→2)-*O*-*α*-l- arabinopyranosyl-28-*O*-*β*-d-xylopyranosyl-(1→6)-*O*-*β*-d-glucopyranosyl ester (**2**) [14], Hedragenin -3-*O*-*α*-l-arabinopyranosyl-28-*O*-*α*-l-rhamnopyranosyl-(1→2)-*O*-*β*-d-glucopyranosyl ester (**3**) [15], Loniceroside A (**4**), Loniceroside B (**5**), Hederagenin-28-*O*-*β*-d-glucopyranosyl-(1→6)-*O*-*β*-d- xylopyranosyl ester (**6**), Hederagenin-3-*O*-*α*-l-arabinopyranosyl-28-*O*-*β*-d-glucopyranosyl-(1→6)- *O*-*β*-d-xylopyranosyl ester (**7**), Hederagenin-28-*O*-*α*-*l*-rhamnopyranosyl-(1→2)-[*O*-*β*-d-xylopyrano- syl-(1→6)]-*O*-*β*-d-glucopyranosyl ester (**8**) [16], Loniceroside C (**9**) [17], Loniceroside D (**10**) [18], Akebiasaponin F (**11**), Hederagenin-3-*O*-*α*-l-rhamnopyranosyl-(1→2)-*O*-*α*-l-arabinopyranosyl-28- *O*-*β*-d-glucopyranosyl ester (**12**), Hederagenin-3-*O*-*α*-l-rhamnopyranosyl-(1→2)-*O*-*α*-l-arabinopy- ranosyl-28-*O*-6-acetyl-*β*-d-glucopyranosyl-(1→6)-*O*-*β*-d-glucopyranoside (**13**) [19], Hederagenin-3- *O*-*β*-d-glucopyranosyl-28-*O*-*β*-d-glucopyranosyl ester (**14**), Hederagenin-3-*O*-*β*-d-glucopyranosyl- (1→2)-*O*-*β*-d-glucopyranosyl-28-*O*-*β*-d-glucopyranosyl-(1→6)-*O*-*β*-d-glucopyranosyl ester (**15**), Hederagenin-3-*O*-*α*-l-rhamnopyranosyl-(1→2)-*O*-*α*-l-arabinopyranosyl-28-*O*-*β*-d-glucopyra-nosyl-(1→6)-*O*-*β*-d-xylopyranosyl ester (**16**) [20] and Hederagenin-3-*O*-*α*-l-rhamnopyranosyl-(1→2)-*O*-*β*- d-xylopyranosyl ester (**17**) [21] have been isolated from *l. japonica* Thunb. species. Hederagenin- 28-*O*-*β*-d-glucopyranosyl-(1→6)-*O*-*β*-d-glucopyranosyl ester (**18**), *α*-hederin (**19**), Macranthoside A (**20**), Macranthoside B (**21**) [22], Dipsacoside B (**22**), Macranthoidin A (**23**) and Macranthoidin B (**24**) [23] have been obtained from *L. confuse* DC. species. Hederagenin-3-*O*-*β*-d-glucopyranosyl-(1→3)-*O*- *α*-l-rhamnopyranosyl-(1→2)-*O*-*α*-l-arabinopyranosyl-23-*O*-acetyl-28-*O*-*β*-d-glucopyranosyl-(1→6)-*O*-*β*-d-glucopyranosyl ester (**25**), Cauloside C (**26**), HN-Saponin F (**27**) [24], Akebiasaponin D (**28**) [25], Lonimacranthoide I (**29**) [26], Lonimacranthoide III (**30**) [27], Lonimacranthoide IV (**31**), Lonimacranthoide V (**32**) [28], Macranthoidin C (**33**), Hederagenin-3-*O*-*α*-l-rhamnnopyranosyl- (1→2)-*α*-l-arabinopyranosyl-28-*O*-*α*-l-rhamnopyransyl-(1→4)-*O*-*β*-d-glucopyranosyl-(1→6)-*O*-*β*-d-glucopyranosyl ester (**34**), Cauloside D (**35**), Hederagenin-3-*O*-*β*-d-glucopyranosyl-(1→4)-*O*-*α*-l- arabinopyranosyl-28-*O*-*α*-l-rhamnopyranosyl-(1→4)-*O*-*β*-d-glucopyranosyl-(1→6)-*O*-*β*-d-glucopyranosyl ester (**36**) and Hederagenin-3-*O*-*β*-d-glucopyranosyl-(1→3)-*O*-*α*-l-rhamnopyranosyl-(1→2)- *O*-*α*-l-arabinopyranosyl-28-*O*-*α*-l-rhamnopyranosyl-(1→4)-*O*-*β*-d-glucopyranosyl-(1→6)-*O*-*β*-d-glucopyranosyl ester (**37**) [29] have been isolated from *L. macranthoides* Hand.-Mazz. species. Sapindoside B (**38**), Fulvotomentoside A (**39**) [30] and Fulvotomentoside B (**40**) [31] have been given from *L. fulvotomentosa* Hsu et S.C. Cheng species. Hederagenin-3-*O*-*β*-d-glucopyranosyl-(1→2)- *O*-*β*-d-glucopyranosyl ester (**41**), Hederagenin-3-*O*-*α*-d-ribosyl-(1→3)-*O*-*α*-l-rhamnopyranosyl- (1→2)-*O*-*α*-l-arabinopyranoside (**42**) and Hederagenin-3-*O*-*β*-d-glucopyranosyl-(1→4)-*O*-*α*-d- ribosyl-(1→3)-*O*-*α*-l-rhamnopyranosyl-(1→2)-*O*-*α*-l-arabinopyranoside (**43**) [32] have been acquired from *L. nigra*.L. species. Hederagenin-3-*O*-*β*-d-glucuronopyranoside (**44**) [33], Hederagenin (**45**) [34] and Hederagenin-3-*O*-*β*-d-xylopyranosyl-(1→3)-*O*-*α*-l-rhamnopyranosyl-(1→2)-*O*-*α*-l-arabinopy- ranosyl-28-*O*-*β*-d-glucopyranosyl-(1→6)-*O*-(3-*O*-caffeoyl)-*β*-d-glucopyranosyl ester (**46**) [35] have been isolated from *L. nigru* L., *L. maackii* Maxim. and *L. dasystyla* Rehd. species, respectively.

### 2.2. Oleanane-Type Triterpenoid Saponins

So far, 17 Oleanane-type triterpenoid saponins (**47**–**63**) have been found from *L. japonica* Thunb., *L. macranthoides* Hand.-Mazz., *L. maackii* Maxim. and *L. nigru* L. species and their structures are listed in Figure 1 and Table 2. 28-*O*-*α*-l-rhamnopyranosyl-(1→2)-[*O*-*β*-d-xylopyranosyl-(l→6)]-*O*-*β*-d- glucopyranosyl-oleanolic acid (**47**) [13], Loniceroside E (**48**) [18], 3-*O*-*α*-l-arabinopyranosyl-28- *O*-*β*-d-glucopyranosyl-(1→6)-*O*-*β*-d-glucopyranosyl-oleanolic acid (**49**), 3-*O*-*β*-d-glucopyranosyl- (1→2)-*O*-*α*-l-arabinopyranosyl-oleanolic acid (**50**), 3-*O*-*β*-d-glucopyranosyl-(1→2)-*O*-*α*-l- arabinopyranosyl-28-*O*-*β*-d-glucopyranosyl-(1→6)-*O*-*β*-d-glucopyranosyl-oleanolic acid (**51**), 3-*O*- *α*-l-rhamnopyranosyl-(1→2)-*O*-*α*-l-arabinopyranosyl-oleanolic acid (**52**) [19], 3-*O*-*α*-l-rhamnopy- ranosyl-(1→2)-*O*-*α*-l-arabinopyranosyl-28-*O*-*β*-d-glucopyranosyl-(1→6)-*O*-*β*-d-glucopyranosyl-oleanolic acid (**53**) [36] and 3-*O*-acetyl-oleanolic acid (**54**) [37] have been isolated from *L. japonica* Thunb. species. Lonimacranthoide II (**55**), 3-*O*-*β*-d-glucopyranosyl-(1→3)-*O*-*α*-l-rhamnopyranosyl-(1→2)- *O*-*α*-l-arabinopyranosyl-28-*O*-*β*-d-glucopyranosyl-(1→6)-*O*-*β*-d-glucopyranosyl-oleanolic acid (**56**) [27] and 3-*O*-*α*-l-rhamnopyranosyl-(1→2)-*O*-*α*-l-arabinopyranosyl-28-*O*-*α*-l-rhamnopyranosyl- (1→4)-*O*-*β*-d-glucopyranosyl-(1→6)-*O-β*-d-glucopyranosyl-oleanolic acid (**57**) [29] have been obtained from *L. macranthoides* Hand.-Mazz. species. Oleanolic acid (**58**) [34], 3*β*-Hydroxyurs- 12-en-28-oic acid ethyl ester (**59**) [38], 3*β*-Hydroxyolean-12-en-27-oic acid (**60**), 3*β*-Hydroxyolean- 12-en-27-oic acid ethyl ester (**61**) [39] and Erythrodiol (**62**) [40] have been given from *L. maackii* Maxim. species. Androseptoside A (**63**) [33] has been isolated from *L. nigru* L. species.

### 2.3. Ursane-Type Triterpenoid Saponins

Thus far, Ursolic acid (**64**) [41], Ziyuglycoside II (**65**) [42], Uvaol (**66**) [39] and Ursolic alcohol (**67**) [40] have been isolated from *L. japonica* Thunb., *L. hypoglauca* Miq. and *L. maackii* Maxim. species, respectively. Their structures are shown in Figure 1 and Table 3.

### 2.4. Lupane-Type Triterpenoid Saponins

Until now, eight Lupane-type triterpenoid saponins have been found from *Lonicera Linn.* and their structures are listed in Figure 1 and Table 4. Bourneioside A–E (**68–72**) [43,44] have been isolated from *L. bournei* Hemsl. species. Lonisimilioside A (**73**), Lonisimilioside C (**74**) and Lonisimilioside D (**75**) [45] have been given from *L. similis* Hemsl. species.

### 2.5. Fernane-1-Type and Fernane-2-Type Triterpenoid Saponins

At present, three Fernane-1 and two Fernane-2-type triterpenoid saponins (**76–80**) [46] have been isolated from *L. gracilipes* var. glandulosa Maxim. species; the structures of Ferna-7,9(11)- diene-3*α*,16*α*-diol (**76**), 3*α*,16*α*-dihydroxyferna-7,9(11)-dien-12-one (**77**), Ferna-7,9(11)-diene- 3*α*,16*α*,19*α*-triol (**78**), 3*α*,16*α*-dihydroxyfern-8-en-11-one (**79**) and 3*α*,16*α*-dihydroxyfern-8-en-7,11- dione (**80**) are shown in Figure 1 and Table 5.

### 2.6. Other Triterpenoid Saponins

**Figure 2:**
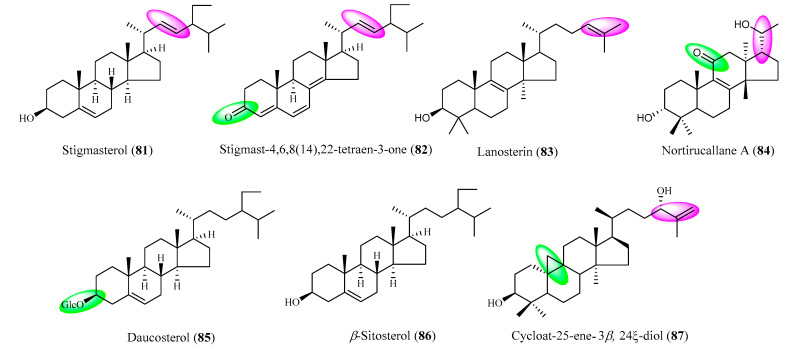
The structures of others triterpenoid saponins compounds (**81**–**87**).

Other chemical constituents, except for Hederin-type, Oleanane-type, Ursane-type, Lupane-type, Fernane-1-type, and Fernane-2-type triterpenoid saponins, were also found in *Lonicera Linn*., the structures of which are shown in Figure 2. Stigmasterol (**81**), Stigmast-4,6,8(14),22- tetraen-3-one (**82**), Lanosterin (**83**) [37], and Nortirucallane A (**84**) [47] have been isolated from *L. japonica* Thunb. species. Daucosterol (**85**) [39], *β*-Sitosterol (**86**) and Cycloart-25-ene-3*β*,24*ξ*-diol (**87**) [48] have been obtained from *L. maackii* Maxim. and *L. saccata* Rehd. species, respectively.

## 3. Biological Activities

**Figure 3:**
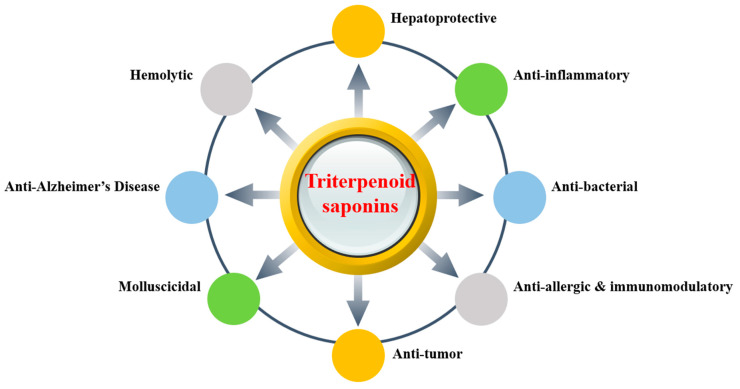
The bioactivities schematic of triterpenoid saponins.

It is visible that the active triterpenoid saponins compounds of *Lonicera Linn*. play a crucial role in biological activities and pharmacological applications. It has been found that they have diverse activities, including hepatoprotective, anti-inflammatory, anti-bacterial, anti-allergic, immunomodulatory, anti-tumor, molluscicidal, and anti-alzheimer’s disease activities, and hemolytic toxicity. These bioactivities are closely related to the traditional effect of “treating carbuncle and furuncle, mitigating swelling, curing pharyngitis, erysipelas, heat toxin blood dysentery, wind-heat type common cold and febrile disease” in Chinese Pharmacopoeia. That is why the triterpenoid saponins of *Lonicera Linn*. have been gaining extensive attention. The bioactivities schematic of triterpenoid saponins are shown in Figure 3.

### 3.1. Hepatoprotective Effect

At present, some saponins from the genus *Lonicera Linn.* are attracting more and more attention because of their hepatoprotective effect. Fulvotomentosides (Ful, the total saponins of *Lonicera fulvotomentosa* Hsu et S.C. Cheng) could significantly reduce the levels of serum glutamic pyruvic transaminase (SGPT) and triacylglycerol (GT) in mice poisoned by CCl_4_, d-galactosamine (d-gal) and acetaminophen (AA), and obviously reduce the pathological damage of liver [49].

AA could be metabolized by liver cytochrome P-450 in vivo, and it produced toxic intermediate N-acetyl-p-benzoquinone imine (NAPQI), which may form a complex with intrahepatic GSH and be detoxified through urine [50]. When excessive AA appeared in the body and the intrahepatic glutathione (GSH) was exhausted, the covalent binding with NAPQI and hepatocyte protein lead to hepatocyte necrosis [51]. *α*-hederin (**19**) and Sapindoside B (**38**) have played a major role in the hepatoprotective effect. The mixture of **19** and **38** could increase the content of GSH in mice, which enhances the detoxification function of liver to AA and reduces the damage of liver. Meanwhile, the protective mechanism was that glucuronidation increased the detoxification of AA and cytochrome P-450 inhibited the toxic activity of AA [52].

Components **19** and **38** could inhibit P450 enzymes activity in mice when used alone or in combination, which may reduce the active metabolites and alleviate toxic damage. This effect is reversible. In the same way, the mixture of **19** and **38** also could reduce the P-450 enzymes activity induced by phenobarbital [49].

CCl_4_, a widely used experimental hepatotoxicant, was biotransformed by cytochrome P-450 system to produce the trichloromethyl free radical, which could engender covalent binding with membranes and organelles to elicit lipid peroxidation and disturb Ca^2+^ homeostasis, leading to cell death [53]. Ful could decrease the increase of malondialdehyde (MDA) caused by CCl_4_, and markedly reduce the hepatotoxicity of CCl_4_ and *D*-gal [54]. Li, et al. [55] indicated that the total saponins of *L. japonica* Thunb. have a significant protective effect against CCl_4_-induced acute liver injury. Oleanolic acid (**58**), a triterpenoid extracted from *L. maackii* Maxim., has protective effect on acute liver injury and chronic cirrhosis induced by CCl_4_, and is used to treat human hepatitis [54]. At the same time, it also could effectively prevent liver injury induced by AA in mice.

Cadmium (Cd), an environmental pollutant, could cause serious liver damages by increasing the activities of serum alanine aminotransferase (ALT/GPT) and sorbitol dehydrogenase (SDH), and produce widespread liver congestion and necrosis [56]. Ful may protect the liver from Cd hepatotoxicity by inducing the liver to synthesize a large number of Metallothionein (MT), and the MT could combine with Cd in the cytoplasm. Thereby, it will reduce the distribution of Cd in the nucleus, mitochondria, microsomes, and cytoplasm of the polymer proteins, and decrease the toxicity of Cd in liver cells. The protective effect of Ful (150 mg/kg) on liver injury induced by Cd is more obvious than caused by CCl_4_, *D*-gal and AA [56].

### 3.2. Anti-Inflammatory and Anti-Bacterial Effects

The published reports have shown that some saponins of the genus *Lonicera Linn*. possess anti-inflammatory and anti-bacterial effect. KWAK, et al. [17] found that Loniceroside A (**4**) and Loniceroside C (**6**) have anti-inflammatory effect on mouse ear edema caused by croton oil. The anti-inflammatory activity of **4** was comparable to aspirin at a dose of 100 mg/kg [57]. Hederagenin (**45**), an aglycone of **4**, also could show anti-inflammatory activity in the same model, and reduce the arthritis induced by adjuvant in rats (100 mg/kg/day). These findings demonstrated that **4** exhibited anti-inflammatory activity against acute and chronic inflammation, and **45** possessed anti-inflammatory and anti-arthritic activities in rats. Oleanolic acid (**59**) has been shown to possess anti-inflammatory, immunomodulatory, and anti-tumor promotion in skin and antiulcer effects [54].

Liu, et al. [58] reported that the Ful could reduce the number of cells in BALF and the activity of myeloperoxidase (MPO), and down-regulate the expression of inflammatory factors ICAM-1, P-selectin, IL-6 and TNF-*α* in BALF and serum, and improve the inflammatory infiltration of lung tissue in mice, whilst also down-regulating the expression of complement factor C5a and C5b-9 in lung tissue. Meanwhile, the Ful could improve the acute lung injury induced by cobra venom factor, and reduce the inflammatory response in mice. The Ful (100 μg/mL) could down-regulate the expression of NF-κB p65 protein, and reduce the ratio of Bax/Bcl-2. The mechanism may be related to NF-κB and JAK2 signaling pathway and involved in the regulation of oxidative stress-induced injury process.

The increased expression of IL-6 and IL-17A was the characteristic of ovalbumin (OVA) sensitized intestinal inflammatory reaction in BALB/c mice [59]. The Ful could reduce the overexpression of IL-6 and IL-17A, and increase the expression of specific transcription factor Foxp3 of CD4^+^, CD25^+^ regulatory T cells in intestine. It may be the mechanism that Ful could improve intestinal inflammation.

Liu, et al. [60] found that the Ful has significant inhibitory effect on foot swelling induced by carrageenan in rats, it could inhibit the increase of capillary permeability produced by various inflammatory agents, but it has no anti-inflammatory effect on adrenalectomy rats. So, the anti-inflammatory effect was achieved by promoting the release of adrenocortical hormone.

Cauloside A (**1**), *α*-hederin (**19**), Dipsacoside B (**22**) and Sapindoside B (**38**) were shown a strong anti-bacterial activity against Gram-positive bacteria *staphylococcus aureus*, *staphylococcus epidermidis*, and Gram-negative bacteria *pseudomonas aeruginosa*, *escherichia coli*, *enterobacter cloacae* and *klebsiella pneumoniae* (MIC values 1.80~2.50 μg/mL) [61]. The anti-bacterial effect of those compounds was similar to Netilmicin, and the activities of **38** and **22** in some cases were even better.

### 3.3. Anti-Allergic and Immunomodulatory Effects

The anti-allergic and immunomodulatory activities of saponins from the *Lonicera Linn.* genus were often discussed together. The high concentration of Ful has a significant inhibitory effect on footpad swelling reaction and OVA-specific IgE in serum, and it could inhibit aggregation and degranulation of mast cells in jejunum and mesentery [62]. The Ful has anti-allergic effect on OVA-sensitized BALB/c mice, it could treat both IgE and non-IgE-mediated food allergy. Moreover, after treatment with the Ful, the levels of OVA-specific IgE and IL-4 were decreased, and the percentage of CD4^+^, CD25^+^, and Foxp3^+^ regulatory T cells and the ratio of IFN-γ/IL-4 were increased. So, the Ful could induce CD4^+^, CD25^+^, and Foxp3^+^ regulatory T cells; enhance Treg reaction; weaken Th2 reaction in spleen; improve Th1/Th2 imbalance; and alleviate IgE mediated hypersensitivity [63].

After inhalation of specific antigen in food allergy mice, the expression of TGF-*β*1, IL-6, and IL-17A were markedly increased in lung, which caused a serious inflammatory reaction of neutrophil infiltration [64]. The Ful could decrease the expression of IL-6, IL-17A to some extent.

Hederagenin-28-*O*-*β*-d-glucopyranosyl-(1→6)-*O*-*β*-d-glucopyranosyl ester (**18)**, Macranthoidin A (**23**), HN-Saponin F (**27**), Akebiasaponin D (**28**), and Hederagenin (**45**) have strong anticomplement activity in the classical activation pathway of complement. The complement system was a major effector of the humoral immunity involved in the host defense. Compound **27** has the most potent anticomplementary activity, followed by compound **28**. It indicated that the C-28 monodesmosidic saponins and C-3, 28 bidesmosidic saponins of hederagenin have an anticomplementary activity [65].

### 3.4. Anti-Tumor Effect

Up to now, the research has detected that saponins from the *Lonicera Linn.* genus exhibit significant anti-tumor activity. Macranthoside B (**21**) could inhibit the proliferation of various cancer cells with IC_50_ values in the range of 10~20 μM. After HepG2 cells were treated with **21** for 4 h, the Caspase-3 was activated due to the expression of procaspase-9 having decreased significantly, and the protein level of Caspase-3 p17 and p12 subunits increased [66]. The expression of Bcl-2 was decreased and the level of Bax was increased, leading to the increase of the Bax/Bcl-2 ratio. The compound **21** was involved in the regulation of mitochondrion-mediated apoptosis pathway, and it could inhibit the proliferation and growth of HepG2 cells in xenograft tumors in athymic BALB/c nude mice. Furthermore, compound **21**, the monodesmosidic saponin with a free carboxyl at C-28, has significant cytotoxic activities against HepG2, MCF-7 and A-549 cell lines with IC_50_ values of 8.98 ± 0.19, 12.48 ± 0.45 and 11.62 ± 0.54 μM, respectively [45]. At the concentrations of 2.5, 5 and 10 μM, it has obvious morphological changes in HepG2 cells, and showed typical apoptotic phenomena with chromatin condensation and karyopyknosis. Similarly, compound **21** could significantly induce apoptosis on A-549 cells.

Cauloside A (**1**), *α*-hederin (**19**), and Sapindoside B (**38**) have significant inhibitory activity on leukemia K562 cells with IC_50_ values of 4, 9 and 11 μM, respectively [61]. Therefore, the presence of a free carboxy group was important for the cytotoxic activity.

Akebiasaponin D (**28**) has strong cytotoxicity against U937 human leukemia cells. It could increase the subG1 cell population and the expression of p53 and Bax, and also enhance NO production from RAW264.7 macrophage cells [67]. Compound **28** could induce apoptosis and play an anti-tumor role by activating NO and the expression of apoptosis-related p53, Bax. Macranthoidin C (**33**), Hederagenin-3-*O*-*α*-l-rhamnnopyranosyl-(1→2)-*α*-l-arabinopyranosyl-28-*O*- *α*-l-rhamnopyransyl-(1→4)-*O*-*β*-d-glucopyranosyl-(1→6)-*O*-*β*-d-glucopyranosyl ester (**34**), and Cauloside D (**35**) showed cytotoxicities against HeLa cells with IC_50_ values of 54.3, 43.9 and 61.2 μmol/L, respectively [56]. Lonimacranthoide I (**30**), a natural complex saponin with novel structure, was composed of triterpenoid saponins and chlorogenic acid. It has significant inhibitory effect on tumor metastasis target matrix metalloproteinase-9 (MMP-9) and cyclooxygenase-2 (COX-2), with IC_50_ values of 11.2 and 2.2 μmol/L, respectively [68]. The chlorogenic acyl group was the key group of anti-tumor activity.

### 3.5. Molluscicidal Effect

Nowadays, some saponins obtained from this genus also have been confirmed to show molluscicidal effect. Cauloside A (**1**) and *α*-hederin (**19**) showed remarkable molluscicidal activities, with the minimum active concentrations required to kill the snails of 5.4 and 6.2 μg/mL, respectively [61]. The toxic concentration of Sapindoside B (**38**) to the snails was 12.8 μg/mL, and the presence of a free carboxy at C-17 was crucial. Meanwhile, Gopalsamy, et al. [69] revealed that compounds **1**, **19** and **26**, three monodesmosidic saponins, possessed significant molluscicidal activity against the schistosomiasis-transmitting snails *Biomphaluria glabrata*. Huang, et al. [33] also investigated that Hederagenin-3-*O*-*β*-d-glucuronopyranoside (**44**), Loniceroside E (**48**) and Androseptoside A (**63**) have strong molluscicidal activity against snails, among them, compound **48** has the highest activity and killed the snails at a concentration of 2 ppm within 24 h. So, bidesmosidic saponins were usually only weakly active or inactive against snails, but their monodesmosidic saponins derivatives were active.

### 3.6. Anti-Alzheimer’s Disease Effect

In the most recent years, the triterpenes saponins from this genus have been proven to exhibit anti-Alzheimer’s Disease (AD) properties. Senile plaques of AD patients were composed primarily of the overaccumulation of *β*-amyloid peptide (A*β*). Akebiasaponin D (**28**), the important compound in saponins fraction, has significant neuroprotective capacity to antagonize Aβ_25-35_-induced cytotoxicity in PC 12 cells. The protective effect was mediated by blocking Aβ-induced Ca^2+^-intake, LDH release and preventing the loss of cell viability and cell apoptosis [70]. Compound **28** may be a promising active component in the treatment of AD.

### 3.7. Hemolytic Toxicity

Recently, more and more researchers have carried out some investigations on saponins’s hemolytic toxicity. Saponins were generally considered to possess hemolytic toxicity [19]. Saponins could form complexes with sterols of the erythrocyte membrane, and increase permeability and leakage of hemoglobin in the cell, thus causing hemolysis [61]. Wang, et al. [14] suggested that monodesmosidic saponins, such as Cauloside A (**1**), *α*-hederin (**19**), Cauloside C (**26**), and Loniceroside E (**48**), showed obvious hemolytic toxicity. Compound **1** evoked over 90% hemolysis at 30 μg/mL, which has a strong hemolytic toxicity regardless of the number of sugars. The presence of a free carboxylic acid and attachment of sugars at C-3 were responsible for the hemolytic toxicity [65]. Compounds **26** and **48** have glucopyranosyl-arabinopyranosyl moiety at C-3; they revealed very strong hemolytic toxicity. There was no significant difference in the toxicity between oleanolic acid saponins and hederagenin saponins.

Bidesmosidic saponins, such as Hederagenin-28-*O*-*α*-l-rhamnopyranosyl-(1→2)-[*O*-*β*-d- xylopyranosyl-(1→6)]-*O*-*β*-d-glucopyranosyl ester (**8**), Loniceroside C (**9**), Loniceroside D (**10**) and Dipsacoside B (**22**), showed relatively weak toxicity. Akebiasaponin D (**28**) and 3-*O*-*α*-l- arabinopyranosyl-28-*O*-*β*-d-glucopyranosyl-(1→6)-*O*-*β*-d-glucopyranosyl-oleanolic acid (**49**) have arabinopyranosyl moiety at C-3 and glucopyranosyl moiety at C-28, which showed no hemolytic toxicity [19]. Thus, monodesmosidic saponins showed strong hemolytic toxicity, but bisdesmosidic showed weak hemolytic toxicity.

## 4. Conclusions

Medicinal and edible plants are usually considered as edible plants that can be used as Chinese medicinal materials to prevent and cure diseases. The application of medicinal and edible plants has a long history in China. Plants of the genus *Lonicera Linn.* have proven to be used in functional foods, cosmetics and other applications, such as *L. japonica* Thunb. *L. japonica* Thunb., as a kind of traditional Chinese medicine for both medicine and food, and can be used to make herbal tea, toothpaste and so on. However, certain aspects still need to be further explored.

In this review, the triterpenoid saponins chemical constituents and biological activities studies of the genus *Lonicera Linn.* were systematically summarized. Currently, 87 saponins and sapogenin chemical components have been isolated from this genus, and they are composed of 46 Hederin-type triterpenoid saponins, 17 Oleanane-type triterpenoid saponins, 4 Ursane-type triterpenoid saponins, 8 Lupane-type triterpenoid saponins, 3 Fernane-1-type triterpenoid saponins, 2 Fernane-2-type triterpenoid saponins, and 7 other compounds.

In regard to biological activities of these triterpenoid saponins chemical constituents, the genus *Lonicera Linn.* has received increasing attention all over the world. Modern pharmacological studies have suggested that triterpenoid saponins constituents have a number of diverse and complex biological activities, including hepatoprotective, anti-inflammatory, anti-bacterial, anti-allergic immunomodulatory, anti-tumor, molluscicidal, and anti-alzheimer’s disease activities, and hemolytic toxicity. Amongst these broad-ranging properties, hemolytic toxicity should be paid more attention.

This review expounds the chemical ingredients and its bioactivities of *Lonicera Linn.* genus, which may not only contribute to the scientific understanding of the traditional application, but also benefit the new drug research and product development of *Lonicera Linn.* genus.

## Figures and Tables

**Table 1:** The structures of Hederin-type triterpenoid saponins compounds (**1**–**46**).

No.	Name	R_1_	R_2_	R_3_	Sp.	Ref.
**1**	Cauloside A	Ara	H	H	*L. japonica* Thunb.	[13]
**2**	Hederagenin-3-*O*-*α*-l-rhamnopyranosyl-(1→2)-*O*-*α*-l-arabinopyranosyl-28-*O*-*β*-d-xylopyranosyl-(1→6)-*O*-*β*-d-glucopyranosyl ester	Rha (1→2) ara	Xyl (1→6) glc	H	*L. japonica* Thunb.	[14]
**3**	Hedragenin-3-*O*-*α*-*L*-arabinopyranosyl-28-*O*-*α*-l-rhamnopyranosyl-(1→2)-*O*-*β*-d-glucopyranosyl ester	Ara	Rha (1→2) glc	H	*L. japonica* Thunb.	[15]
**4**	Loniceroside A	Ara	Rha (1→2) [xyl (1→6)] glc	H	*L. japonica* Thunb.	[16]
**5**	Loniceroside B	Rha (1→2) ara	Rha (1→2) [xyl (1→6)] glc	H	*L. japonica* Thunb.	[16]
**6**	Hederagenin-28-*O*-*β*-d-glucopyranosyl-(1→6)-*O*-*β*-d-xylopyranosyl ester	H	Glc (1→6) xyl	H	*L. japonica* Thunb.	[16]
**7**	Hederagenin-3-*O*-*α*-l-arabinopyranosyl-28-*O*-*β*-d-glucopyranosyl-(1→6)-*O*-*β*-d-xylopyranosyl ester	Ara	Glc (1→6) xyl	H	*L. japonica* Thunb.	[16]
**8**	Hederagenin-28-*O*-*α*-l-rhamnopyranosyl-(1→2) [*O*-*β*-d-xylopyranosyl-(1→6)]-*O*-*β*-d-glucopyranosyl ester	H	Rha (1→2) [xyl (1→6)] glc	H	*L. japonica* Thunb.	[16]
**9**	Loniceroside C	Glc	Rha (1→2) [xyl (1→6)] glc	H	*L. japonica* Thunb.	[17]
**10**	Loniceroside D	Glc	Glc (1→2) [xyl (1→6)] glc	H	*L. japonica* Thunb.	[18]
**11**	Akebiasaponin F	Glc (1→2) ara	Glc (1→6) glc	H	*L. japonica* Thunb.	[19]
**12**	Hederagenin-3-*O*-*α*-l-rhamnopyranosyl-(1→2)-*O*-*α*-l-arabinopyranosyl-28-*O*-*β*-d-glucopyranosyl ester	Rha (1→2) ara	Glc	H	*L. japonica* Thunb.	[19]
**13**	Hederagenin-3-*O*-*α*-l-rhamnopyranosyl-(1→2)-*O*-*α*-l-arabinopyranosyl-28-*O*-6-acetyl-*β*-d-glucopyranosyl-(1→6)-*O*-*β*-d-glucopyranoside	Rha (1→2) ara	Glc (1→6) glc-6-Ac	H	*L. japonica* Thunb.	[19]
**14**	Hederagenin-3-*O*-*β*-d-glucopyranosyl-28-*O*-*β*-d-glucopyranosyl ester	Glc	Glc	H	*L. japonica* Thunb.	[20]
**15**	Hederagenin-3-*O*-*β*-d-glucopyranosyl-(1→2)-*O*-*β*-d-glucopyranosyl-28-*O*-*β*-d-glucopyranosyl-(1→6)-*O*-*β*-d-glucopyranosyl ester	Glc (1→2) glc	Glc (1→6) glc	H	*L. japonica* Thunb.	[20]
**16**	Hederagenin-3-*O*-*α*-l-rhamnopyranosyl-(1→2)-*O*-*α*-l-arabinopyranosyl-28-*O*-*β*-d-glucopyranosyl-(1→6)-*O*-*β*-d-xylopyranosyl ester	Rha (1→2) ara	Glc (1→6) xyl	H	*L. japonica* Thunb.	[20]
**17**	Hederagenin-3-*O*-*α*-l-rhamnopyranosyl-(1→2)-*O*-*β*-d-xylopyranosyl ester	Rha (1→2) xyl	H	H	*L. japonica* Thunb.	[21]
**18**	Hederagenin-28-*O*-*β*-d-glucopyranosyl-(1→6)-*O*-*β*-d-glucopyranosyl ester	H	Glc (1→6) glc	H	*L. confuse* DC.	[22]
**19**	*α*-hederin	Rha (1→2) ara	H	H	*L. confuse* DC.	[22]
**20**	Macranthoside A	Glc (1→3) rha (1→2) ara	H	H	*L. confuse* DC.	[22]
**21**	Macranthoside B	Glc (1→4) glc (1→3) rha (1→2) ara	H	H	*L. confuse* DC.	[22]
**22**	Dipsacoside B	Rha (1→2) ara	Glc (1→6) glc	H	*L. confuse* DC.	[23]
**23**	Macranthoidin A	Glc (1→3) rha (1→2) ara	Glc (1→6) glc	H	*L. confuse* DC.	[23]
**24**	Macranthoidin B	Glc (1→4) glc (1→3) rha (1→2) ara	Glc (1→6) glc	H	*L. confuse* DC.	[23]
**25**	Hederagenin-3-*O*-*β*-d-glucopyranosyl-(1→3)-*O*-*α*-l-rhamnopyranosyl-(1→2)-*O*-*α*-l-arabinopyranosyl-23-*O*-acetyl-28-*O*-*β*-d-glucopyranosyl-(1→6)-*O*-*β*-d-glucopyranosyl ester	Glc (1→3) rha (1→2) ara	Glc (1→6) glc	Ac	*L. macranthoides* Hand.-Mazz.	[24]
**26**	Cauloside C	Glc (1→2) ara	H	H	*L. macranthoides* Hand.-Mazz.	[24]
**27**	HN-Saponin F	Ara	Glc	H	*L. macranthoides* Hand.-Mazz.	[24]
**28**	Akebiasaponin D	Ara	Glc (1→6) glc	H	*L. macranthoides* Hand.-Mazz.	[25]
**29**	Lonimacranthoide I	Glc (1→4) glc (1→3) rha (1→2) ara	Glc (1→6) glc	Chlorogenic acyl	*L. macranthoides* Hand.-Mazz.	[26]
**30**	Lonimacranthoide III	Glc (1→4) glc (1→3) rha (1→2) ara	Glc	H	*L. macranthoides* Hand.-Mazz.	[27]
**31**	Lonimacranthoide IV	Glc (1→3) rha (1→2) ara	Glc	Chlorogenic acyl	*L. macranthoides* Hand.-Mazz.	[28]
**32**	Lonimacranthoide V	Glc (1→4) glc (1→3) rha (1→2) ara	Glc (1→6) [(4-*O*-sulfo)] glc	H	*L. macranthoides* Hand.-Mazz.	[28]
**33**	Macranthoidin C	Glc (1→4) ara	Glc	H	*L. macranthoides* Hand.-Mazz.	[29]
**34**	Hederagenin-3-*O*-*α*-l-rhamnnopyranosyl-(1→2)-*α*-l-arabinopyranosyl-28-*O*-*α*-l-rhamnopyransyl-(1→4)-*O*-*β*-d-glucopyranosyl-(1→6)-*O*-*β*-d-glucopyranosyl ester	Rha (1→2) ara	Rha (1→4) glc (1→6) glc	H	*L. macranthoides* Hand.-Mazz.	[29]
**35**	Cauloside D	Ara	Rha (1→4) glc (1→6) glc	H	*L. macranthoides* Hand.-Mazz.	[29]
**36**	Hederagenin-3-*O*-*β*-d-glucopyranosyl-(1→4)-*O*-*α*-l-arabinopyranosyl-28-*O*-*α*-l-rhamnopyranosyl-(1→4)-*O*-*β*-d-glucopyranosyl-(1→6)-*O*-*β*-d-glucopyranosyl ester	Glc (1→4) ara	Rha (1→4) glc (1→6) glc	H	*L. macranthoides* Hand.-Mazz.	[29]
**37**	Hederagenin-3-*O*-*β*-d-glucopyranosyl-(1→3)-*O*-*α*-l-rhamnopyranosyl-(1→2)-*O*-*α*-l-arabinopyranosyl-28-*O*-*α*-l-rhamnopyranosyl-(1→4)-*O*-*β*-d-glucopyranosyl-(1→6)-*O*-*β*-d-glucopyranosyl ester	Glc (1→3) rha (1→2) ara	Rha (1→4) glc (1→6) glc	H	*L. macranthoides* Hand.-Mazz.	[29]
**38**	Sapindoside B	Xyl (1→3) rha (1→2) ara	H	H	*L. fulvotomentosa* Hsu et S.C. Cheng	[30]
**39**	Fulvotomentoside A	Xyl (1→3) rha (1→2) ara	Glc (1→4) glc	H	*L. fulvotomentosa* Hsu et S.C. Cheng	[30]
**40**	Fulvotomentoside B	Xyl (1→3) rha (1→2) ara	Xyl (1→6) glc	H	*L. fulvotomentosa* Hsu et S.C. Cheng	[31]
**41**	Hederagenin-3-*O*-*β*-d-glucopyranosyl-(1→2)-*O*-*β*-d-glucopyranosyl ester	Glc (1→2) glc	H	H	*L. nigra*. L.	[32]
**42**	Hederagenin-3-*O*-*α*-d-ribosyl-(1→3)-*O*-*α*-l-rhamnopyranosyl-(1→2)-*O*-*α*-l-arabinopyranoside	Rib (1→3) rha (1→2) ara	H	H	*L. nigra*. L.	[32]
**43**	Hederagenin-3-*O*-*β*-d-glucopyranosyl-(1→4)-*O*-*α*-d-ribosyl-(1→3)-*O*-*α*-l--rhamnopyranosyl-(1→2)-*O*-*α*-l-arabinopyranoside	Glc (1→4) rib (1→3) rha (1→2) ara	H	H	*L. nigra*. L.	[32]
**44**	Hederagenin-3-*O*-*β*-d-glucuronopyranoside	Glc	H	H	*L. nigru* L.	[33]
**45**	Hederagenin	H	H	H	*L. maackii* Maxim.	[34]
**46**	Hederagenin-3-*O*-*β*-d-xylopyranosyl-(1→3)-*O*-*α*-l--rhamnopyranosyl-(1→2)-*O*-*α*-l-- arabinopyranosyl-28-*O*-*β*-d-glucopyranosyl-(1→6)-*O*-(3-*O*-caffeoyl)-*β*-d-glucopyranosyl ester	Xyl (1→3) rha (1→2) ara	Glc (1→6) [(3-*O*-caffeoyl)] glc	H	*L. dasystyla* Rehd.	[35]

**Table 2:** The structures of Oleanane-type triterpenoid saponins compounds (**47**–**63**).

No.	Name	R_1_	R_2_	R_3_	Sp.	Ref.
**47**	28-*O*-*α*-l-rhamnopyranosyl-(1→2)-[*O*-*β*-d-xylopyranosyl-(l→6)]-*O*-*β*-d-glucopyranosyl-oleanolic acid	H	COO-rha (1→2) [xyl (1→6)] glc	CH_3_	*L. japonica* Thunb.	[13]
**48**	Loniceroside E	Glc	COO-rha (1→2) [xyl (1→6)] glc	CH_3_	*L. japonica* Thunb.	[18]
**49**	3-*O*-*α*-l-arabinopyranosyl-28-*O*-*β*-d-glucopyranosyl-(1→6)-*O*-*β*-d-glucopyranosyl- oleanolic acid	Ara	COO-glc (1→6) glc	CH_3_	*L. japonica* Thunb.	[19]
**50**	3-*O*-*β*-d-glucopyranosyl-(1→2)-*O*-*α*-l-arabinopyranosyl-oleanolic acid	Glc (1→2) ara	COOH	CH_3_	*L. japonica* Thunb.	[19]
**51**	3-*O*-*β*-d-glucopyranosyl-(1→2)-*O*-*α*-l-arabinopyranosyl-28-*O*-*β*-d-glucopyranosyl-(1→6)-*O*-*β*-d-glucopyranosyl-oleanolic acid	Glc (1→2) ara	COO-glc (1→6) glc	CH_3_	*L. japonica* Thunb.	[19]
**52**	3-*O*-*α*-l-rhamnopyranosyl-(1→2)-*O*-*α*-l-arabinopyranosyl-oleanolic acid	Rha (1→2) ara	COOH	CH_3_	*L. japonica* Thunb.	[19]
**53**	3-*O*-*α*-l-rhamnopyranosyl-(1→2)-*O*-*α*-l-arabinopyranosyl-28-*O*-*β*-d-glucopyranosyl-(1→6)-*O*-*β*-d-glucopyranosyl-oleanolic acid	Rha (1→2) ara	COO-glc (1→6) glc	CH_3_	*L. japonica* Thunb.	[36]
**54**	3-*O*-acetyl-oleanolic acid	COCH_3_	COOH	CH_3_	*L. japonica* Thunb.	[37]
**55**	Lonimacranthoide II	Glc (1→4) glc (1→3) rha (1→2) ara	COO-glc (1→6) glc	CH_3_	*L. macranthoides* Hand.-Mazz.	[27]
**56**	3-*O*-*β*-d-glucopyranosyl-(1→3)-*O*-*α*-l-rhamnopyranosyl-(1→2)-*O*-*α*-l-arabinopyranosyl-28-*O*-*β*-d-glucopyranosyl-(1→6)-*O*-*β*-d-glucopyranosyl-oleanolic acid	glc (1→3) rha (1→2) ara	COO-glc (1→6) glc	CH_3_	*L. macranthoides* Hand.-Mazz.	[27]
**57**	3-*O*-*α*-l-rhamnopyranosyl-(1→2)-*O*-*α*-l-arabinopyranosyl-28-*O*-*α*-l-rhamnopyranosyl-(1→4)-*O*-*β*-d-glucopyranosyl-(1→6)-*O-β*-d-glucopyranosyl-oleanolic acid	Rha (1→2) ara	COO-rha (1→4) glc (1→6) glc	CH_3_	*L. macranthoides* Hand.-Mazz.	[29]
**58**	Oleanolic acid	H	COOH	CH_3_	*L. maackii* Maxim.	[34]
**59**	3*β*-Hydroxyurs-12-en-28-oic acid ethyl ester	H	COOC_2_H_5_	CH_3_	*L. maackii* Maxim.	[38]
**60**	3*β*-Hydroxyolean-12-en-27-oic acid	H	CH_3_	COOH	*L. maackii* Maxim.	[39]
**61**	3*β*-Hydroxyolean-12-en-27-oic acid ethyl ester	H	CH_3_	COOC_2_H_5_	*L. maackii* Maxim.	[39]
**62**	Erythrodiol	H	CH_2_OH	CH_3_	*L. maackii* Maxim.	[40]
**63**	Androseptoside A	Glc	COOH	CH_3_	*L. nigru* L.	[33]

**Table 3:** The structures of Ursane-type triterpenoid saponins compounds (**64**–**67**).

No.	Name	R_1_	R_2_	Sp.	Ref.
**64**	Ursolic acid	H	COOH	*L. japonica* Thunb.	[41]
**65**	Ziyuglycoside II	Ara	COOH	*L. hypoglauca* Miq.	[42]
**66**	Uvaol	H	CH_2_OH	*L. maackii* Maxim.	[39]
**67**	Ursolic alcohol	CH_2_OH	COOH	*L. maackii* Maxim.	[40]

**Table 4:** The structures of Lupane-type triterpenoid saponins compounds (**68–75**).

No.	Name	R_1_	R_2_	R_3_	Sp.	Ref.
**68**	Bourneioside A	Glc	Glc	OH	*L. bournei* Hemsl.	[43]
**69**	Bourneioside B	Glc	Glc (1→6) glc	OH	*L. bournei* Hemsl.	[43]
**70**	Bourneioside C	Glc (1→2) glc	Glc (1→6) glc	OH	*L. bournei* Hemsl.	[44]
**71**	Bourneioside D	Glc (1→2) glc (1→6) glc	Glc	OH	*L. bournei* Hemsl.	[44]
**72**	Bourneioside E	Glc (1→2) glc	Glc (1→6) glc	H	*L. bournei* Hemsl.	[44]
**73**	Lonisimilioside A	Glc (1→2) glc	Glc	OH	*L. similis* Hemsl.	[45]
**74**	Lonisimilioside C	Glc (1→2) glc	Glc (1→6) glc	H	*L. similis* Hemsl.	[45]
**75**	Lonisimilioside D	Glc (1→6) glc (1→2) glc	Glc	H	*L. similis* Hemsl.	[45]

**Table 5:** The structures of Fernane-1 and Fernane-2-type triterpenoid saponins compounds (**76**–**80**).

No.	Name	R_1_	R_2_	Sp.	Ref.
**76**	Ferna-7,9(11)-diene-3*α*,16*α*-diol	H_2_	H	*L. gracilipes* var. glandulosa Maxim.	[46]
**77**	3*α*,16*α*-dihydroxyferna-7,9(11)-dien-12-one	O	H	*L. gracilipes* var. glandulosa Maxim	[46]
**78**	Ferna-7,9(11)-diene-3*α*,16*α*,19*α*-triol	H_2_	OH	*L. gracilipes* var. glandulosa Maxim	[46]
**79**	3*α*,16*α*-dihydroxyfern-8-en-11-one	H_2_		*L. gracilipes* var. glandulosa Maxim	[46]
**80**	3*α*,16*α*-dihydroxyfern-8-en-7,11-dione	O		*L. gracilipes* var. glandulosa Maxim	[46]
